# Investigating the Effects of Al_2_O_3_ Microparticles on Wood Waste OSBs: A Study on Physical, Mechanical, and Durability Performance

**DOI:** 10.3390/polym15122652

**Published:** 2023-06-12

**Authors:** Wanley Eduardo Lopes Junior, Matheus Roberto Cabral, André Luis Christoforo, Cristiane Inácio de Campos, Juliano Fiorelli

**Affiliations:** 1 Graduate Program in Materials Science and Engineering, Faculty of Animal Science and Food Engineering, Campus Pirassununga, University of São Paulo, Pirassununga, 13635-900, SP, Brazil; wanley.lopes@usp.br; 2 Natural Sciences and Engineering Research Council of Canada (NSERC), Industrial Research Chair on Eco-Responsible Wood Construction, Department of Wood and Forest Sciences, Université Laval, Quebec City, QC G1V 0A6, Canada; 3 Department of Civil Engineering, Campus São Carlos, Federal University of São Carlos, São Carlos, 13565-905, SP, Brazil; christoforoal@yahoo.com.br; 4 Institute of Sciences and Engineering, Campus of Itapeva, State University of São Paulo Júlio de Mesquita Filho, Itapeva, 18409-010, SP, Brazil; cristiane.campos@unesp.br

**Keywords:** balsa wood, metal oxide, particleboards, polyurethane resin, wood

## Abstract

The development of new materials for the construction sector is a global trend, and products that use by-products in their composition and have also incorporated technology are commercially competitive. Microparticles have large surface areas and can modify the microstructure of materials, positively affecting their physical and mechanical properties. In this context, this study aims to investigate the effect of incorporating aluminium oxide (Al_2_O_3_) microparticles on the physical and mechanical properties of oriented strand boards (OSBs) made from reforested residual balsa and castor oil polyurethane resin and to evaluate their durability performance under accelerated aging conditions. The OSBs were produced on a laboratory scale with a density of 650 kg/m^3^, strand-type particles measuring 90 × 25 × 1 mm^3^, using castor oil-based polyurethane resin (13%) and Al_2_O_3_ microparticle content ranging from 1% to 3% of the resin mass. The physical and mechanical properties of the OSBs were determined following the EN-300:2002 recommendations. The results obtained indicated that the OSBs with 2% Al_2_O_3_ presented thickness swelling significantly lower (at the 5% significance level) after being subjected to accelerated aging and internal bonding of the particles higher than the values obtained for the references, evidencing the positive effect of including Al_2_O_3_ microparticles in balsa OSBs.

## 1. Introduction

In recent years, much research has been conducted on the use of fibrous raw materials from agro-industrial by-products or forest biomass to produce particleboards. These particleboards are bonded using either organic (i.e., resins) or inorganic matrices (i.e., cement) and have shown promising results, suggesting their potential application in various areas such as building elements and furniture. Then, this approach adds value to waste materials and offers a more sustainable alternative for utilizing these raw resources, thereby contributing to environmental conservation and waste reduction efforts [1,2]. Balsa (*Ochroma pyramidale*) wood waste is a noteworthy by-product known for its low density, ranging from 120 kg/m^3^ to 200 kg/m^3^. It is also known for its rapid growth rate, with height reaching 18 to 25 m in approximately 7 years. Furthermore, it possesses favorable mechanical properties, including a modulus of elasticity (MOE) of up to 8 GPa and a modulus of rupture (MOR) of 70 MPa [3].

Therefore, given its technical properties, balsa wood is an interesting raw material for particleboards, particularly oriented strand boards (OSBs), which are structural materials that require excellent strength and stiffness performance. Furthermore, OSB serves as a viable substitute for plywood, as it can be manufactured from tree scraps and other by-products without necessitating high-quality veneers such as plywood [4]. OSB is produced using thin and long wood strands that are bonded by hot pressing with a thermosetting resin [5]. This material is typically formed by three layers of strands, and in the inner layer, the alignment is perpendicular to the formation direction of the board, while in the outer layers, the alignment is parallel [6]. OSB is a commonly used material in wood frame construction as sheathing for shear walls and as coverings for ceilings. Furthermore, OSB can also serve as structural elements that add rigidity to the construction [7]. However, one of the main challenges faced by particleboards, including OSBs, as construction elements is linked to their performance loss due to moisture and biodegradation [8].

The OSB industry conventionally uses phenol-formaldehyde and melamine resins to manufacture moisture-resistant structural materials. However, these resins can have negative health impacts on humans. To tackle this issue, an alternative is the use of castor oil-based polyurethane resin (PU resin). This alternative offers superior mechanical properties and moisture resistance compared to formaldehyde-based resins. In addition, it is composed of 100% solids and does not emit formaldehyde [9,10].

An additional approach for enhancing the performance of particleboards is to include additives, such as micro and nanoparticles, within the resins. Yildirim et al. [11] state that the addition of nanoscale materials can result in the development of new properties within macroscopic materials. Microparticles of metal oxides are also a viable option for improving the physical and mechanical properties of particleboards. The authors further explain that the smaller size of these particles allows them to penetrate the wood and fill their voids, altering the surface chemistry and providing high protection against moisture. The reduced size of these particles lets them interact effectively with the wood fibers, filling voids and resulting in a more uniform composite, improving the material’s resistance to environmental conditions such as moisture [12]. MingZhu et al. [13] also indicated that including metal-based particles (e.g., metal hydroxides, lamellar double hydroxide, metallic Lewis acid, and metallic organic structures such as boron, phosphorus, and carbon) in wood-based composites is a promising strategy to improve the materials fire and mechanical performance.

Furthermore, including metal oxide nanoparticles or microparticles in particleboards enhances the transmission of heat throughout the material’s thickness during production. It results in a more uniform curing of the resin and improves the materials’ physical and mechanical properties [14]. Kumar et al. [15] argue that adding aluminum oxide (Al_2_O_3_) nanoparticles to medium-density fiberboards (MDF) resulted in numerous improvements, such as better adhesion between the fibers, higher MOR and MOE values, lower thickness swelling (TS), modifications to temperature profiles, and thermal conductivity. Zhang et al. [16] state that adding Al_2_O_3_ nanoparticles to the formaldehyde-free (FF) resin used in plywood increased shear strength and optimized the resin curing. These improvements led to greater manufacturing efficiency and reduced energy costs. Currently, there is a research gap in the study of metal oxide microparticles embedded in OSBs made from reforested residual balsa wood, especially regarding their durability performance. Therefore, this study aims to investigate the effect of incorporating Al_2_O_3_ microparticles on the physical and mechanical properties of OSBs made from reforested residual balsa and PU resin and to evaluate its durability performance under accelerated aging conditions.

## 2. Experimental

### 2.1. Materials

For the development of this research, wood wastes were obtained from reforestation balsa wood (SisGen A4206B8) resulting from industrial processing. The study utilized a bi-component Castor PU resin (viscosity of 167 centipoise) prepared by cold mixing a pre-polymer (component A) with 99.92% solids content and a polyol (component B) with 99.91% solids content (solvent-free). The Al_2_O_3_ microparticles used in this study had a molecular weight of 101.96 u, the presence of heavy metals and iron below 0.03% and 0.005%, respectively, and a particle size of 100 mesh.

### 2.2. Balsa Wood OSB Production

The OSBs were produced with a density of 650 kg/m^3^ and were conformed into three particle layers in a mass ratio of 30:40:30 using 13% of castor oil PU resin [5]. The use of this resin content (13%) is justified due to the properties of balsa wood. Balsa wood has low density, high porosity, and larger vessel diameters compared to those species traditionally used in OSB production (i.e., pine and eucalyptus). A greater volume of wood strands is needed to attain the desired density of 650 kg/m^3^ [5]. The contents of Al_2_O_3_ microparticles ranged from 1 to 3% of the resin mass, following the recommendations of [15,16]. In addition, reference OSBs were also produced without Al_2_O_3_.

**Figure 1:**
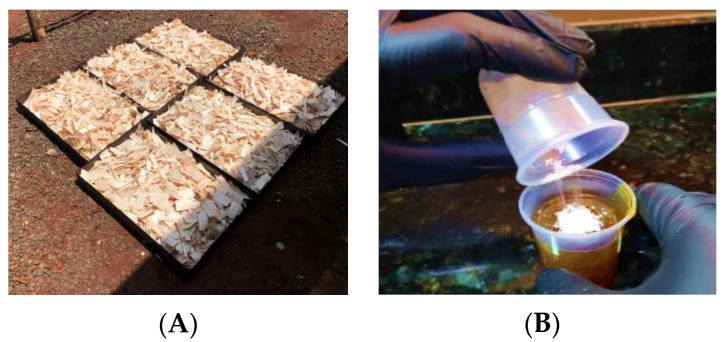

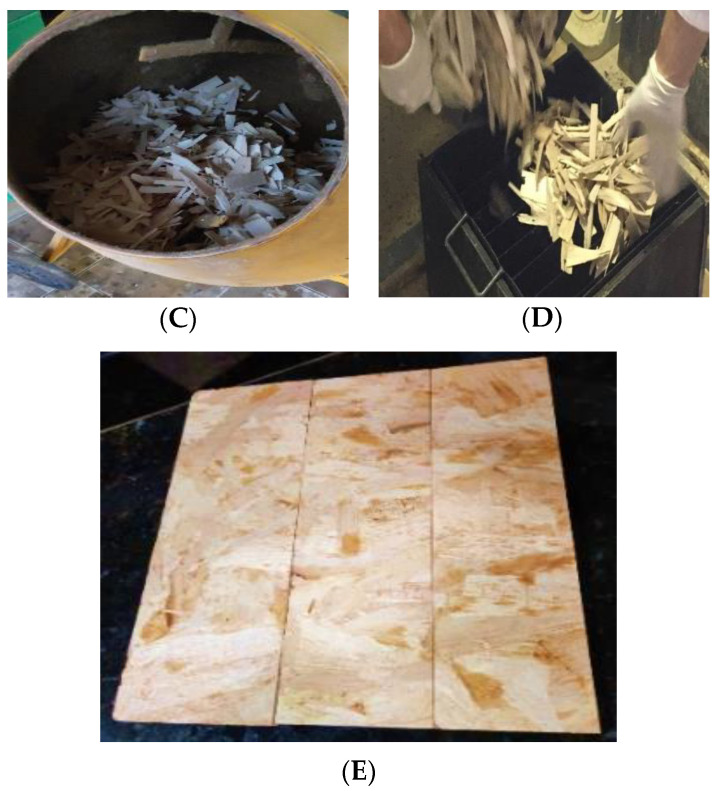
Production of OSBs. (**A**) Balsa wood strands. (**B**) Mixing the resin with microparticles. (**C**) Rotating drum. (**D**) Strands molding. (**E**) Balsa wood OSB.

To produce the OSBs, wood wastes were first transformed into 90 mm long, 25 mm wide, and 1 mm thick strands (Figure 1A) using an electrically driven chipper (Marconi manufacturer, model MA685, Piracicaba, SP, Brazil). These strands were then dried in an oven at 65 °C for 48 h until the moisture content reached 8% [17]. Subsequently, the resin and the Al_2_O_3_ microparticles (1, 2, and 3% of the resin mass) were prepared (Figure 1B). Next, the mattress was subjected to a pressure of 5 MPa at a temperature of 100 °C for 10 min [5,9,17].

After pressing, the 12 OSBs produced, comprising reference OSBs and those containing microparticles, were conditioned at room temperature for 72 h to allow the resin curing process to complete. Following the completion of the analysis, an additional 4 OSBs (i.e., 2 for reference and 2 for Al_2_O_3_ content with better performance) were produced using the same methods as before and were subjected to an accelerated aging test. The contents of Al_2_O_3_ incorporated in the OSBs are listed in Table 1 and are as follows: Reference; OSB with 1% Al_2_O_3_ (C1-A); OSB with 2% Al_2_O_3_ (C2-A); and OSB with 3% Al_2_O_3_ (C3-A). C2-A* denotes the OSB subjected to the accelerated aging test. 

### 2.3. Balsa Wood OSB Characterization

The mechanical tests to determine the OSBs’ longitudinal (MOR-L and MOE-L), transversal (MOR-T and MOE-T), and internal bonding (IB) properties followed the EN 310 [18] and EN 319 [19] standards and recommendations, respectively. Physical tests such as TS values after 24 h were conducted following the recommendations of EN 317 [20]. The results were then compared with the classification criteria established by EN 300 [21] for evaluating the suitability of the OSBs in civil construction and related applications. 

**Figure 2:**
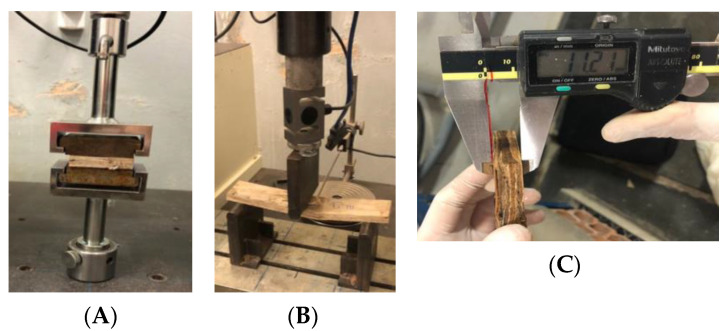
Balsa wood OSB characterization. (**A**) IB test. (**B**) Static bending tests. (**C**) TS test.

The IB test aims to determine the internal adhesion of the particles, and the specimens are square-shaped with (50 ± 1) mm. These specimens were fixed on metal supports, positioned in the device for perpendicular traction testing in the universal EMIC testing machine, and submitted to tensile forces in opposite directions (Figure 2A) at a speed of 4 mm/min.

To determine the MOR and MOE values, specimens measuring 250 × 50 mm^2^ were submitted to three-point static bending tests (span of 220 mm) using a universal testing machine (Figure 2B). Subsequently, the universal machine was calibrated with a sensitivity of 0.1 mm and an accuracy of 1% of the measured value to perform the static flexion test at 3 points. The speed of the test was constant and regulated so that the rupture force was reached in (60 ± 30) s. 

For the TS test, specimens were cut from the boards at 50 × 50 mm. Then, with a Mitutoyo caliper, their thickness sizes were measured before and after their immersion in clean water for 24 h (Figure 2C), with a pH equal to 7 and a temperature of 20 ± 1 °C.

### 2.4. Evaluating the Durability of the OSBs via Accelerated Aging Test

**Figure 3:**
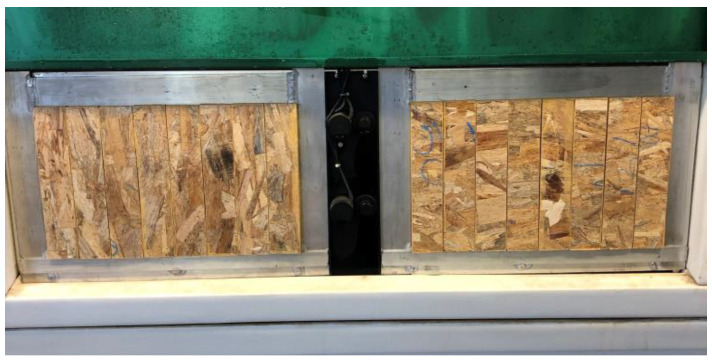
Basal wood OSB specimens in artificial aging chamber.

To evaluate the durability of the OSBs, accelerated aging tests were conducted, as shown in Figure 3. The testing procedure was adapted from APA PRP 108 [22] to simulate the exposure conditions of the OSBs to variations in humidity, temperature, and UVB radiation. The specimens, measuring 250 × 50 × 10 mm (width × depth × height mm), were placed in an artificial UV aging chamber (EQUV—EQUILAN model) and subjected to 8 cycles of 12 h each. During each cycle, the specimens were exposed to 8 h of UVB radiation with 0.49 W/m^2^ irradiance at 60 °C, followed by 4 h of condensation at 50 °C. These conditions simulate the equivalent of six months of exposure in a natural environment.

### 2.5. Statistical Analysis

The mechanical and physical properties of the OSBs (reference and those with added Al_2_O_3_ microparticles) were initially evaluated using descriptive statistics to organize the results. Once the descriptive analysis was carried out, the data were subjected to inferential analysis (Tukey’s mean contrast test at a significance level of 5%). This analysis was conducted to determine whether there are significant differences between the mean values (as shown in Table 2) based on the different levels of aluminum oxide microparticles (1%, 2%, and 3%). Table 2 also displays the number of repetitions tested for obtaining the properties and for each of the outlined Al_2_O_3_ contents, resulting in 482 experimental determinations.

From Tukey’s test, A denotes the Al_2_O_3_ content associated with the highest mean value of a certain property, B the Al_2_O_3_ content related to the second highest mean value, and so on, and equal letters imply distinct compositions with statistically equivalent means (at the 5% significance level). The Anderson-Darling test, also at a 5% significance level, was used to assess normality in the distributions of properties. The *p*-value of the test greater than or equal to the significance level implies accepting normality in the distribution, which validates the results obtained from Tukey’s test and the confidence intervals of the mean (95% reliability). The Johnson transformation (variable scales) was used for distributions considered non-normal a priori by the Anderson–Darling test. Subsequently, the influence of Al_2_O_3_ microparticles on the durability of OSBs before and after aging, concerning their TS and IB properties, was investigated. Thus, a complete factorial design 2^2^ was conceived, which resulted in four distinct experimental treatments, which are explained in Table 3.

For the TS property, five specimens were tested for treatments 2 and 10. For the IB property, ten specimens were characterized for each treatment. For analyzing the results, besides the use of Tukey’s test (5% significance) considered in the previous analysis results, the interaction between the two factors pondered was investigated at this stage. According to the ANOVA formulation, a *p*-value lower than the significance level implies considering the interaction effect of the factors in each property evaluated to be significant and not significant otherwise (*p*-value ≥ 0.05). After finishing the analysis, it was feasible to define the most appropriate content of Al_2_O_3_ microparticles for balsa OSB, aiming at the application of this material for construction purposes.

## 3. Results and Discussion

### 3.1. Physical and Mechanical Properties

Table 4 displays the mean value and coefficients of variation (CV) for the physical and mechanical properties of OSBs produced with varying contents of Al_2_O_3_, ranging from 0% (the reference) to 3%. It is noteworthy that the *p*-values of the Anderson–Darling normality test for all properties and compositions exceeded the significance level of 5%, thus confirming the validity of the results obtained from the mean confidence intervals. Furthermore, the values of these properties were compared to the recommended values for OSB type 2 (used for structural purposes in dry environments) as specified in the European normative document EN 300 [21]. In Table 4, the mean results for TS, MOR, MOE, and IB for the reference OSBs and those containing Al_2_O_3_ microparticles are presented.

For the physical property of TS, analyzing the Al_2_O_3_ contents, it was found that the OSBs with 1, 2, and 3%, and the reference, did not present a statistical difference (by sharing A), indicating that they presented equivalent performance. However, while comparing the mean value for this property, it was identified that the OSB with 2% Al_2_O_3_ presented mean values lower than the other boards. The addition of 2% Al_2_O_3_ presented values similar to those found by Lima et al. [23] for OSBs produced with pine and phenolic resin with 0.5% Al_2_O_3_ nanoparticles, indicating a trend of improvement in the board’s performance in contact with water. Regarding the static bending tests, for both MOR-L and MOE-L, including Al_2_O_3_ with contents of 1 to 3% did not promote significant statistical differences (by sharing A), presenting performance equivalent to reference OSB. However, when comparing the mean value for this property, it is possible to identify that the composition with 2% Al_2_O_3_ presented mean values above the other compositions. In the transverse direction, for both the MOR-T and MOE-T, the compositions C2-A and C3-A presented higher mean values than the reference; however, they are alike in performance (by sharing A).

In the perpendicular tensile strength or IB, the best performance, considering the mean value presented, was noted for the boards with 2% Al_2_O_3_, which differ statistically from the reference OSB. This result may indicate that the agglomeration of the particles has benefited from the addition of the microparticles. One of the reasons for this result can be given by the efficiency of commercial aluminum oxide microparticles in the conduction of heat during the production process of OSB panels since the residual balsa wood presents an average apparent density in the range of 200 kg/m^3^ and the panels were produced with a density of 650 kg/m^3^. This results in a panel with a compaction ratio (quotient of panel density by wood density) equal to 3.3, higher than the compaction ratios recommended by Surdi et al. [24].

Although the compositions present a statistical difference, all met the recommendations of standard EN 300 [21], type 2, for mechanical properties. However, for the TS property, the C1-A, C3-A, and reference OSB are higher than those recommended by EN 300 [21]. While analyzing only the mean value obtained for this property, it is also implied that in the limit of TS = 20% recommended in EN 300 [21] for type 2, the C2-A composition (2% Al_2_O_3_) is selected as the most suitable for the production of OSBs with balsa wood for type 2 boards (for structural purposes used in a dry environment).

The results obtained for the OSBs are in agreement with those found by Kumar et al. [15], who evaluated the effects of aluminum oxide (Al_2_O_3_) nanoparticles on the physical, mechanical, and heat transfer properties of MDF. The nanoparticles were added at contents of 0.5% and 1.0% of the dry weight of the wood fibers. The results showed the temperature profile within the board and the thermal conductivity were improved by the addition of Al_2_O_3_ nanoparticles, subsequently increasing the adhesion of the fibers. In addition, the authors attest that the rupture modulus and the elasticity modulus were improved by the addition of Al_2_O_3_ nanoparticles compared to the reference. The Al_2_O_3_ nanoparticles also reduced the values for thickness swelling of the reference OSBs. 

Zhang et al. [16] concluded in their work that Al_2_O_3_ nanoparticles used with the phenol-formaldehyde adhesive in plywood improved the resin’s performance, resulting in an increase of up to 20% in shear strength. Furthermore, the authors showed that the particles incorporated in the adhesive have the ability to accelerate and optimize the resin curing process, which could increase the efficiency of board manufacturing and reduce energy costs in production.

Although there were some statistical differences between the compositions, it is seen that all of them met the mechanical property recommendations of standard EN 300 [21] type 2. Concerning the physical property of TS, the proposed compositions had a positive influence compared to reference OSB; however, they did not reach the minimum recommendation required by EN 300 [21]. When comparing the physical and mechanical properties of the three microparticle compositions with Al_2_O_3_ to the reference, there were no significant differences found. However, analyzing the mean value obtained, the OSB containing 2% oxide was superior to the other compositions and the reference. Therefore, an artificial aging test was conducted to validate the effectiveness of adding 2% Al_2_O_3_ microparticles to balsa wood OSB.

### 3.2. Balsa Wood OSBs after Accelerated Aging Test

Table 5 shows the mean values and coefficients of variation (in parentheses) of the four idealized experimental treatments of the stage after accelerated aging.

Analyzing the physical properties of TS and the mechanical properties of IB, it is seen that the composition with 2% Al_2_O_3_ microparticles, denoted as C2-A, outperforms the reference composition (Table 4). This trend is also evident in the performance of C2-A compared to reference OSB before aging. Upon evaluating the mean physical and mechanical properties after artificial aging, it is seen that the composition with 2% Al_2_O_3_ has TS values lower than the other compositions. While for the IB, the value is higher than the reference. This indicates the effectiveness of the microparticles in improving the degradation resistance of the OSBs. Table 6 presents the results of Tukey’s mean contrast test (5% significance).

From the results in Table 6, it is seen that the statistical analysis conducted before the aging process revealed that including 2% Al_2_O_3_ did not result in significant improvements in the physical and mechanical properties of the analyzed boards. This is because they did not show any significant statistical differences, and their performance was equivalent to that of reference OSB. However, the data obtained after the accelerated aging process demonstrated the effectiveness of incorporating 2% Al_2_O_3_ microparticles in the OSB in the long term. This was evidenced by the significant statistical differences observed in reference OSB and C2-A, with both sharing A but C2-A showing better performance than reference OSB.

## 4. Conclusions

This study found that OSBs produced with balsa wood with a density of 650 kg/m^3^ and 13% castor oil PU resin, including microparticles of Al_2_O_3_, met the mechanical performance requirements set by EN 300/2002 for type 2 OSB. Although the accelerated aging test caused degradation in the boards, the results indicated that the use of 2% Al_2_O_3_ was effective in improving the long-term performance of the product compared to the reference OSB without microparticles.

Reforested balsa wood has significant potential as a raw material for producing OSB with 13% polyurethane resin based on castor oil and 2% microparticles of Al_2_O_3_. The resulting product, with a density of 650 kg/m^3^, can be utilized in civil construction and related fields. Incorporating microparticles of Al_2_O_3_ in the OSB containing balsa wood proved to be an efficient solution for improving the product’s long-term performance under accelerated aging conditions.

## Figures and Tables

**Table 1:** Experimental plan.

Accelerated Aging Test	Al_2_O_3_ Contents	Percentage of Microparticles	Number of OSBs
No	Reference	—	3
No	C1-A	1	3
No	C2-A	2	3
No	C3-A	3	3
Yes	Reference	—	2
Yes	C2-A *	2	2

* Content with better physical and mechanical performance before the accelerated aging test.

**Table 2:** Specimens by Al_2_O_3_ content and by experimental tests.

Al_2_O_3_ Content	TS	Static Bending				IB
	24 h	MOR-L	MOE-L	MOR-T	MOE-T	
Reference	5	7	7	7	7	10
C1-A	5	7	7	7	7	10
C2-A	10	7	7	7	7	10
C3-A	10	7	7	7	7	10

**Table 3:** Treatments of the second experimental phase.

Al_2_O_3_ Content	Aging
Reference	Yes
Reference	No
C2-A	Yes
C2-A	No

**Table 4:** Mean values of the physical and mechanical properties of the balsa wood OSBs.

Al_2_O_3_ Content	TS (%)	Static Bending (MPa)				IB (MPa)
	24 h	MOR-L	MOR-T	MOE-L	MOE-T	
**Reference** **(CV)**	30.77 ^A^ 22.91	27.26 ^A^ 24.99	12.99 ^B^ 24.43	4514 ^A^ 33.54	1280 ^B^ 15.13	0.29 ^B^ 21.58
**C1-A** **(CV)**	38.35 ^A^ 17.37	27.23 ^A^ 14.57	12.40 ^B^ 12.76	4162 ^A^ 11.61	1266 ^B^ 11.66	0.40 ^AB^ 33.08
**C2-A** **(CV)**	26.49 ^A^ 29.28	30.96 ^A^ 30.92	16.77 ^A^ 10.42	4726 ^A^ 23.67	1795 ^A^ 14.34	0.45 ^A^ 33.98
**C3-A** **(CV)**	34.96 ^A^ 41.35	27.33 ^A^ 30.06	16.96 ^A^ 17.95	3858 ^A^ 26.25	1640 ^AB^ 27.65	0.39 ^AB^ 31.12
**EN 300:2002** **Type 2**	20	22	11	3500	1400	0.34

Means followed by different letters in the column differ significantly at 5% by the Tukey’s test. The results of MOR trans and MOE trans.

**Table 5:** TS and IB of specimens before and after accelerated aging tests.

Al_2_O_3_ Content	Aging	TS 24 h (%)	IB (MPa)
Reference	Yes	25.72 (26.77)	0.12 (55.90)
Reference	No	30.77 (22.91)	0.29 (21.58)
C2-A	Yes	14.34 (34.08)	0.18 (45.63)
C2-A	No	26.49 (29.28)	0.45 (33.98)

**Table 6:** Results of Tukey’s mean contrast test.

Properties	Before Aging		After Aging	
	Reference	C2-A	Reference	C2-A
TS 24 h (%)	A	A	A	B
IB	A	A	A	A
	**Reference**		**C2-A**	
	**No Aging**	**Aging**	**No Aging**	**Aging**
TS 24 h (%)	A	A	A	B
IB	A	B	A	B

Equal letters imply different treatments with means statistically equivalent to each other. Different letters imply treatments with significantly different means, with the highest value of the property represented by the letter A and the lowest by the letter B.

## Data Availability

Some or all data, models, or code that support the findings of this study are available from the corresponding author upon reasonable request.
